# Supplementary figures and images for: Phosphorylation of Rec8 cohesin complexes regulates mono-orientation of kinetochores in meiosis I
Source: Life Sci Alliance. 2024 Mar 6;7(5):e202302556. doi: 10.26508/lsa.202302556 (PMC10917647; doi:10.26508/lsa.202302556)

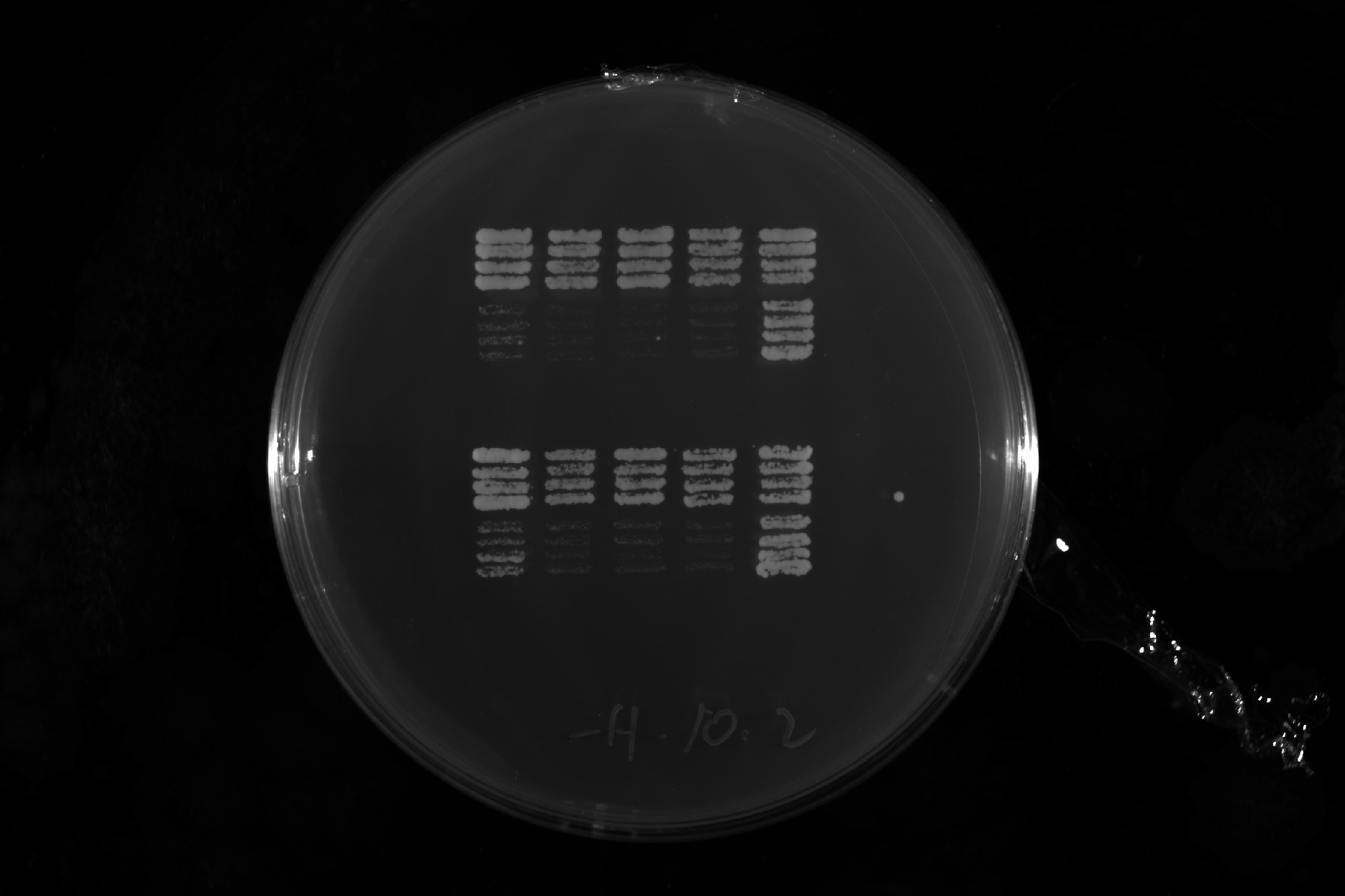

Supplement: Supplementary file 2 [file LSA-2023-02556_SdataF1.2_F2.2_F3.2_F4.2.zip › Raw data-pictures/Fig 2F/1.tif]

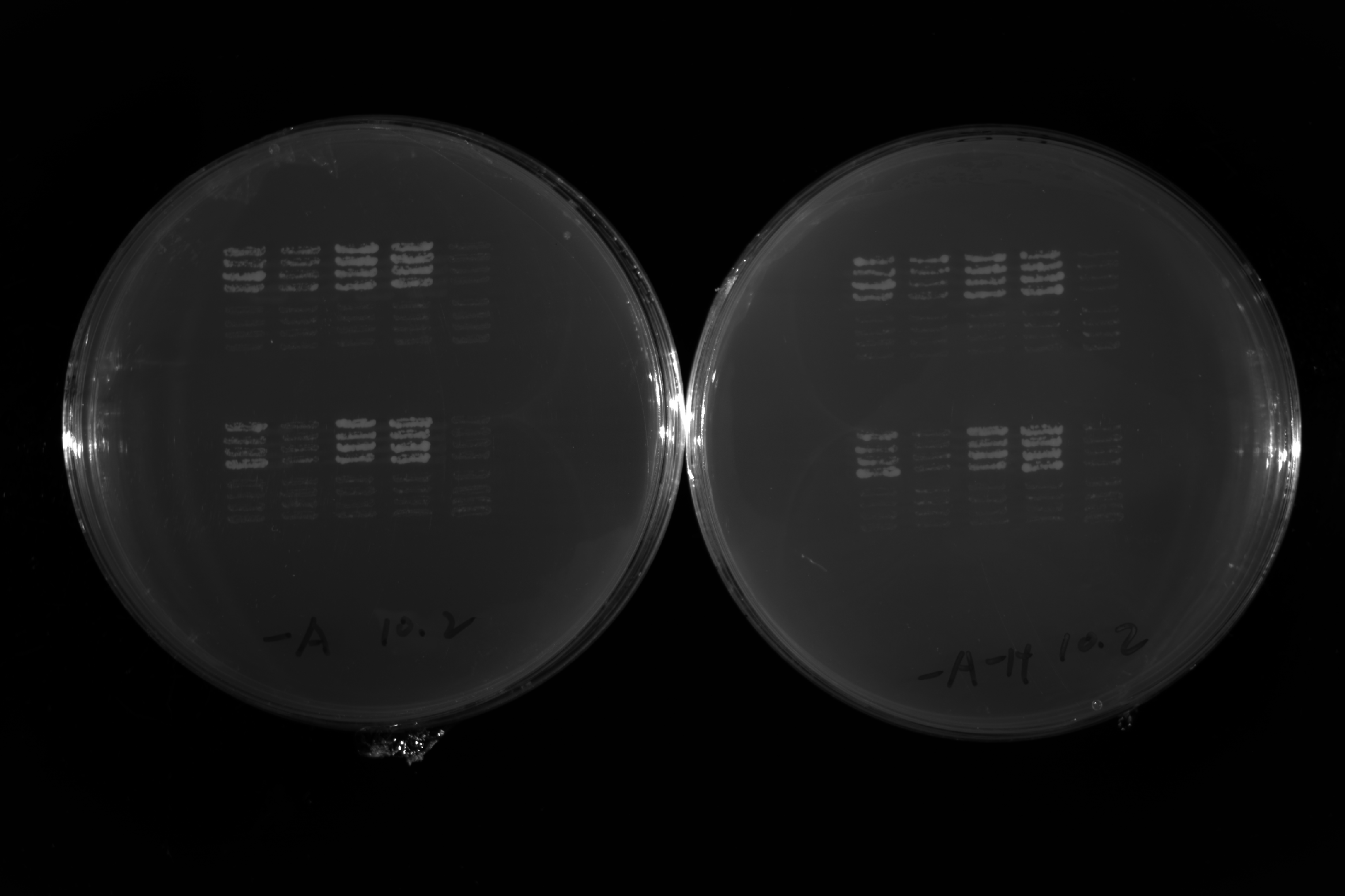

Supplement: Supplementary file 2 [file LSA-2023-02556_SdataF1.2_F2.2_F3.2_F4.2.zip › Raw data-pictures/Fig 2F/2.tif]

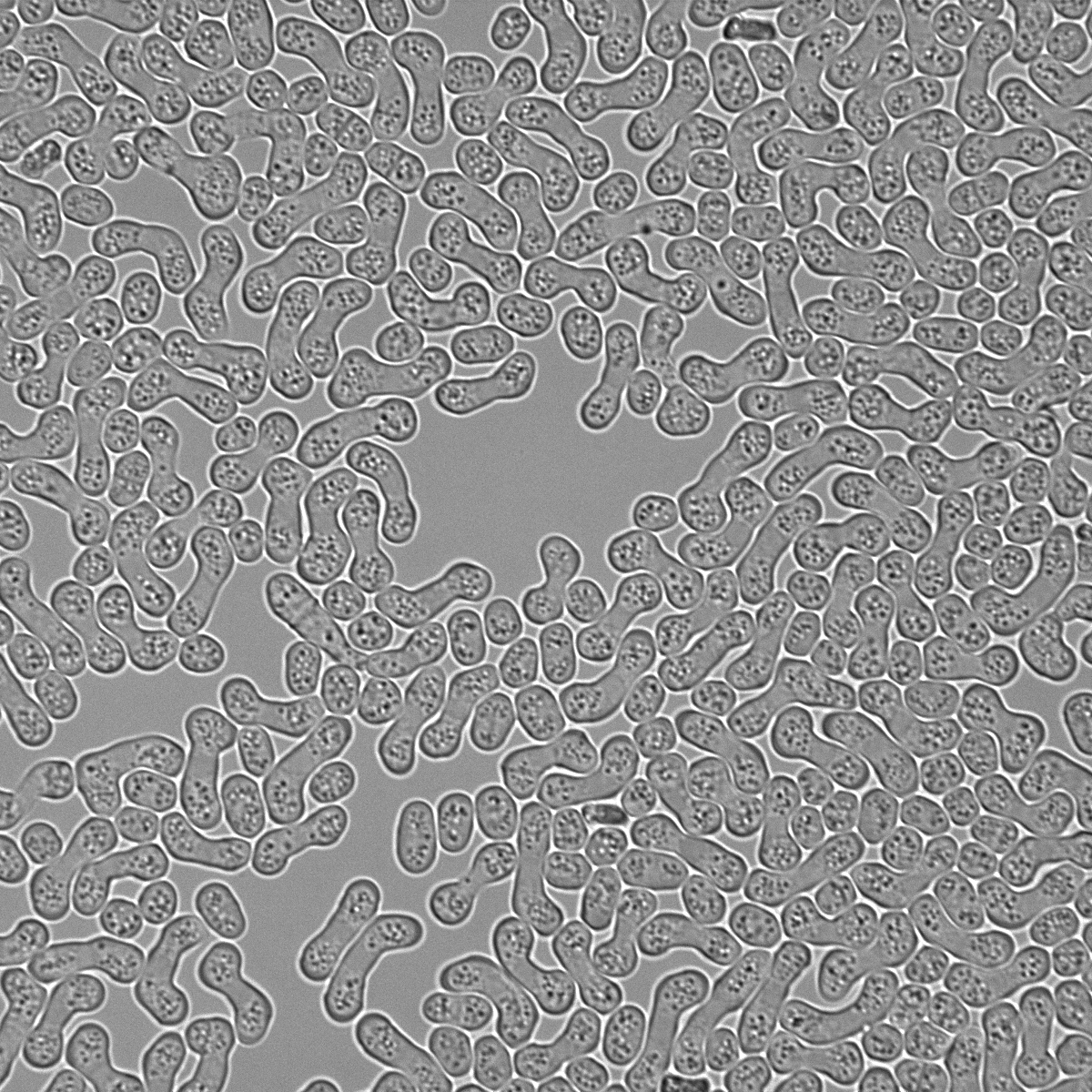

Supplement: Supplementary file 2 [file LSA-2023-02556_SdataF1.2_F2.2_F3.2_F4.2.zip › Raw data-pictures/Fig 3F/psm3-2A/DIC.tif]

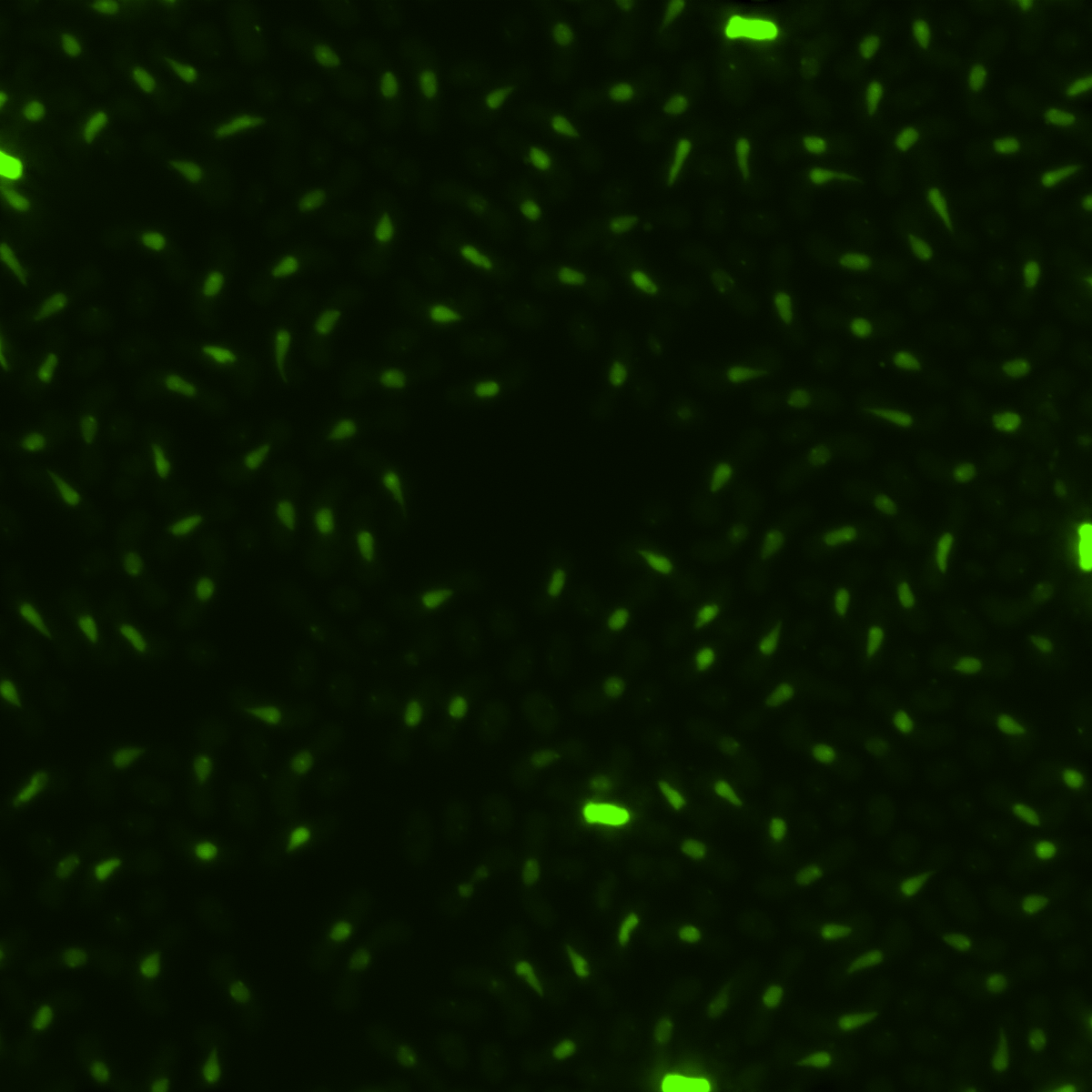

Supplement: Supplementary file 2 [file LSA-2023-02556_SdataF1.2_F2.2_F3.2_F4.2.zip › Raw data-pictures/Fig 3F/psm3-2A/Rec8-GFP.tif]

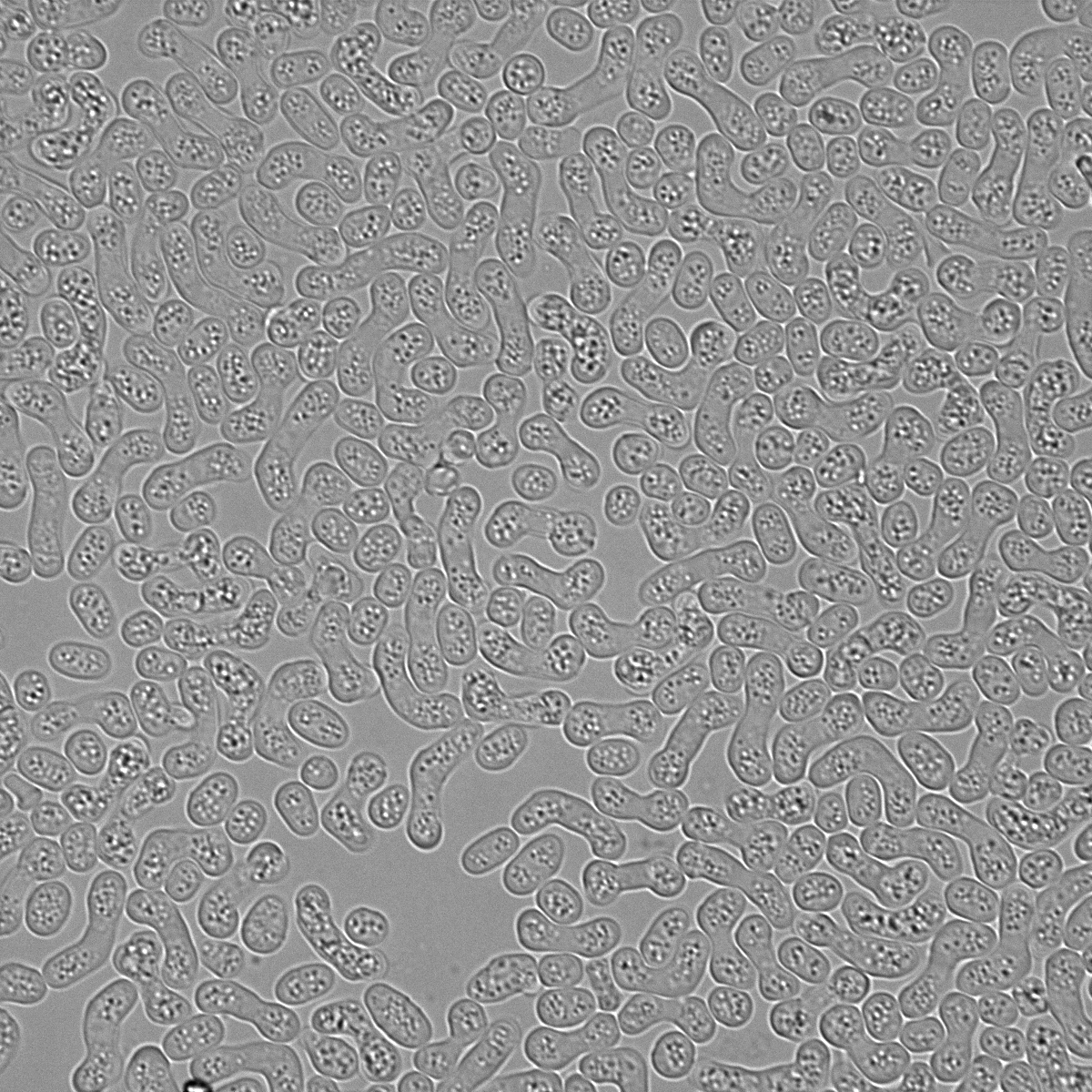

Supplement: Supplementary file 2 [file LSA-2023-02556_SdataF1.2_F2.2_F3.2_F4.2.zip › Raw data-pictures/Fig 3F/psm3-ED/DIC.tif]

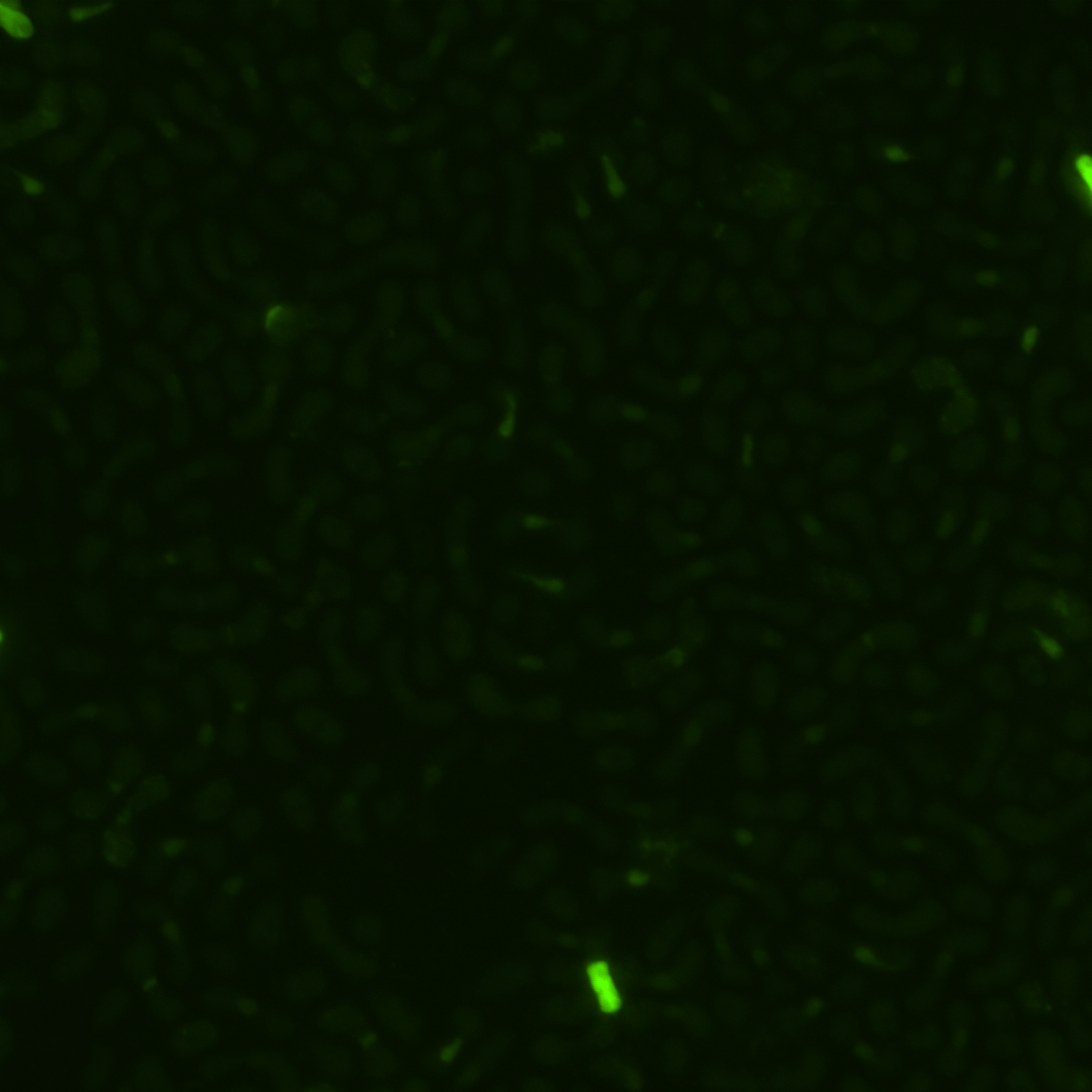

Supplement: Supplementary file 2 [file LSA-2023-02556_SdataF1.2_F2.2_F3.2_F4.2.zip › Raw data-pictures/Fig 3F/psm3-ED/Rec8-GFP.tif]

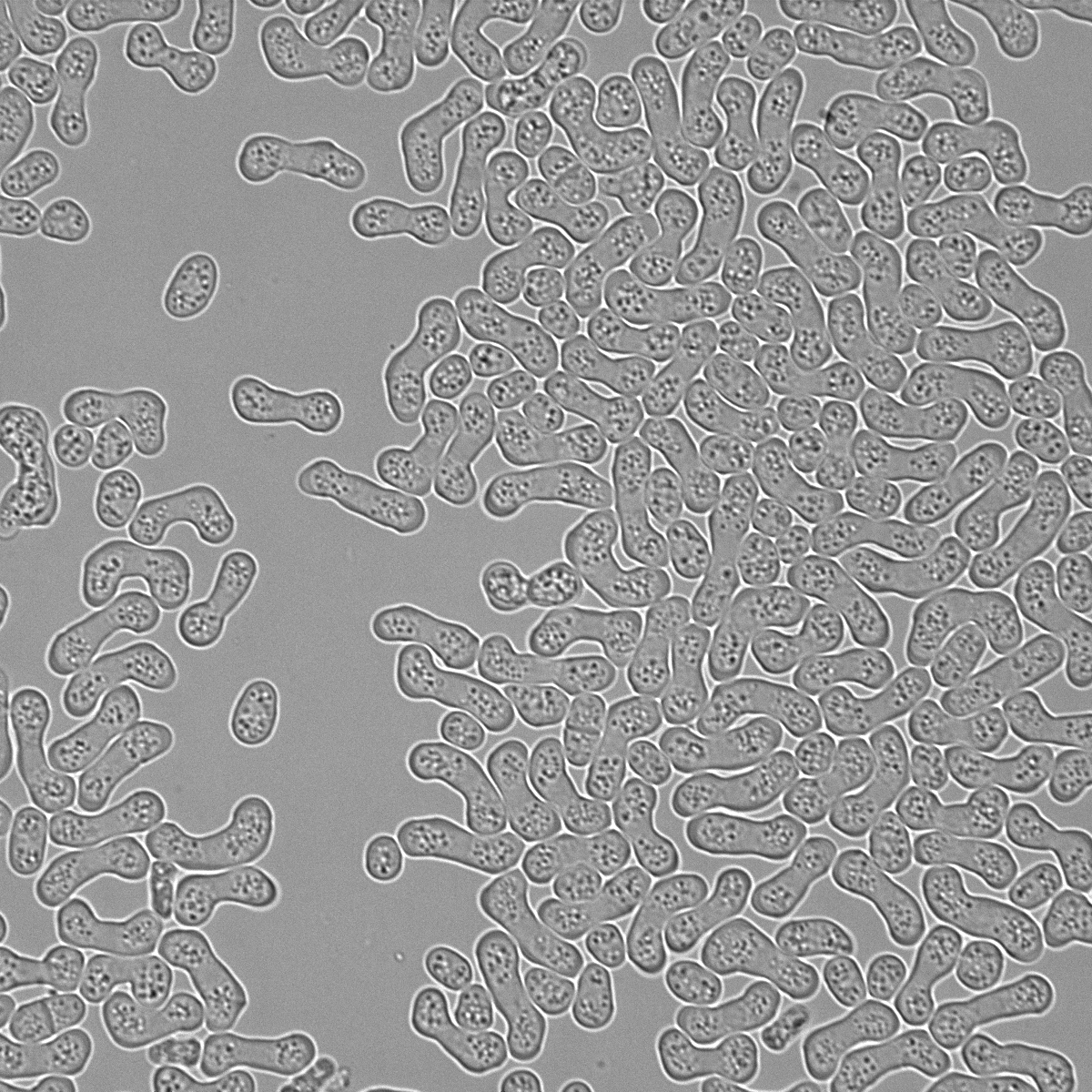

Supplement: Supplementary file 2 [file LSA-2023-02556_SdataF1.2_F2.2_F3.2_F4.2.zip › Raw data-pictures/Fig 3F/wt/DIC.tif]

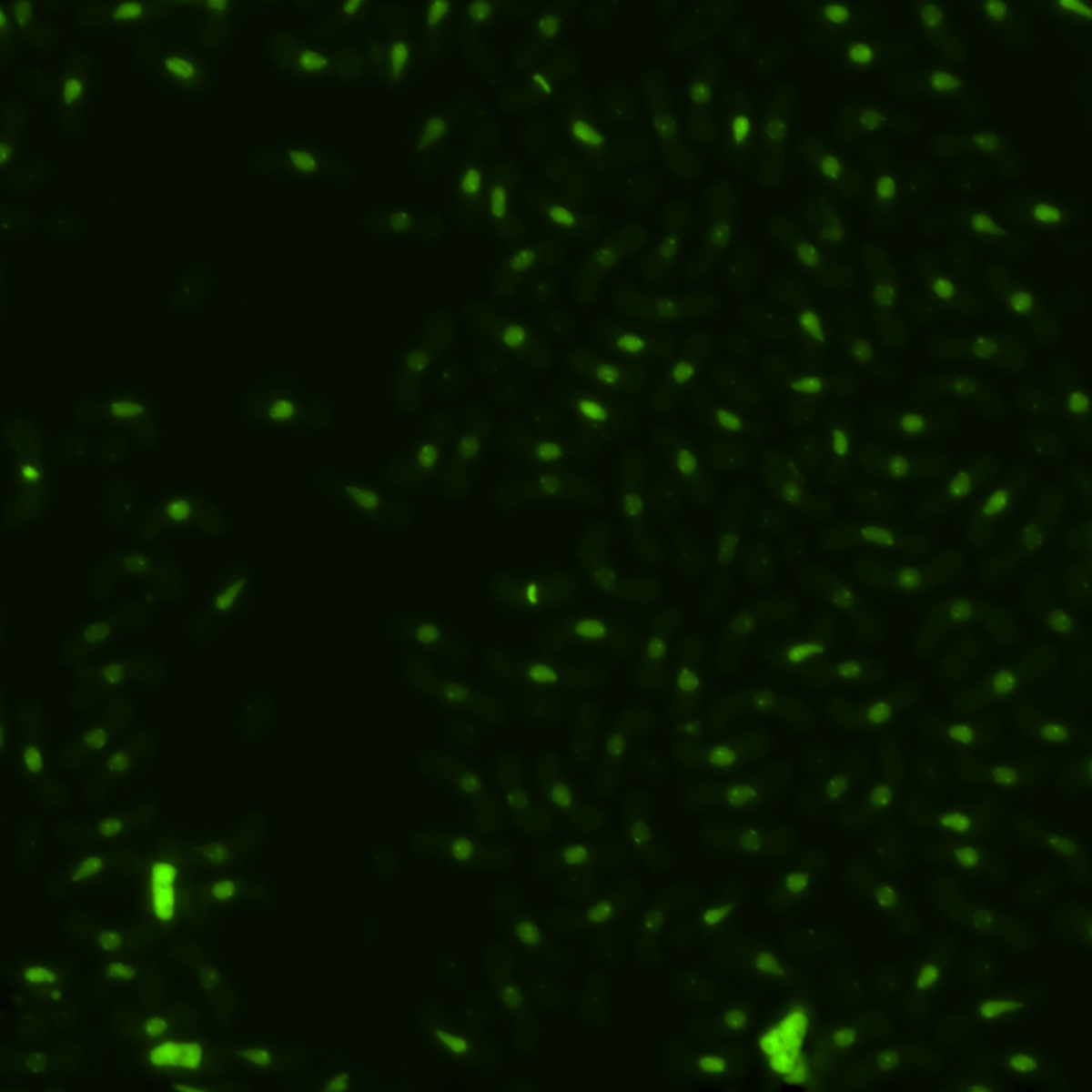

Supplement: Supplementary file 2 [file LSA-2023-02556_SdataF1.2_F2.2_F3.2_F4.2.zip › Raw data-pictures/Fig 3F/wt/Rec8-GFP.tif]

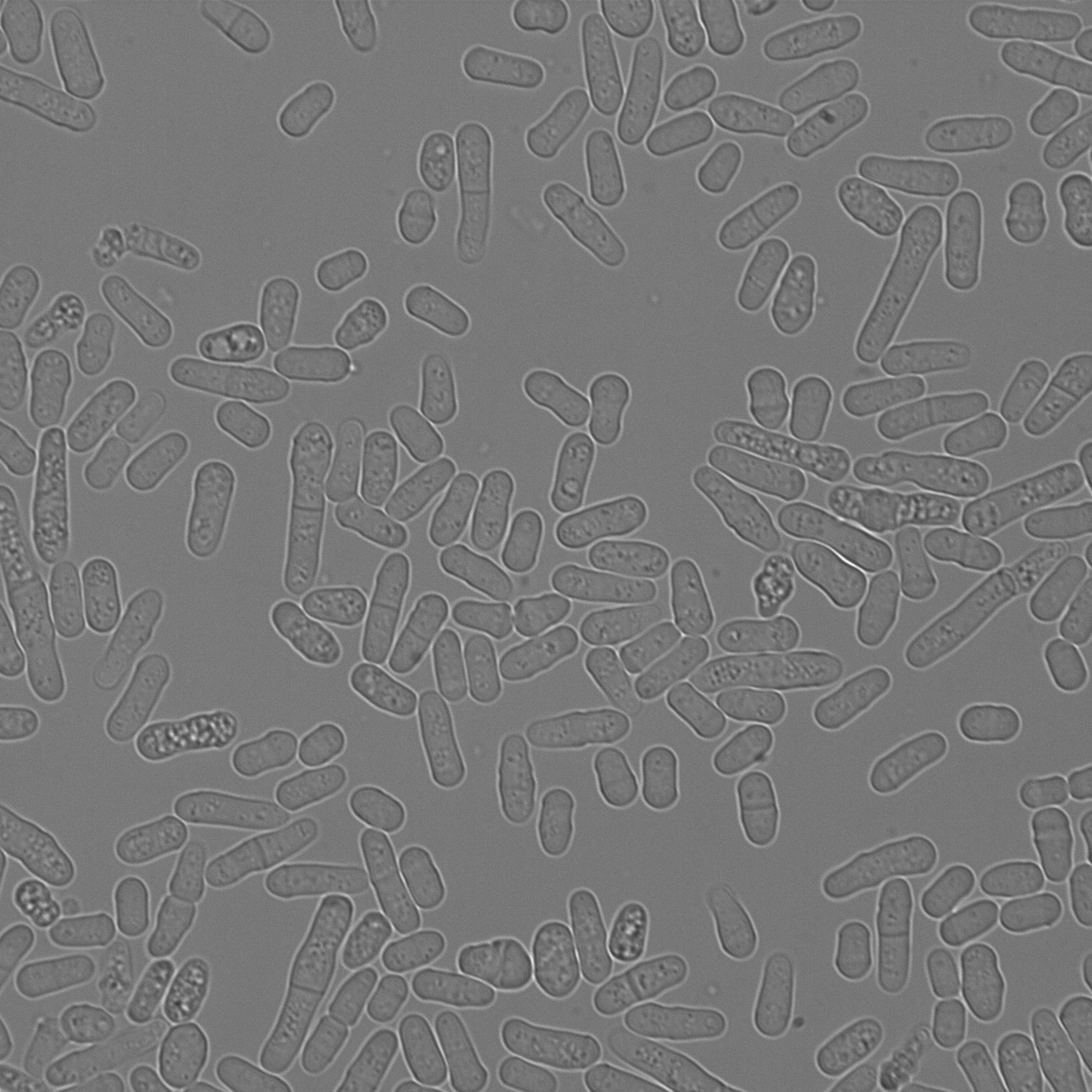

Supplement: Supplementary file 2 [file LSA-2023-02556_SdataF1.2_F2.2_F3.2_F4.2.zip › Raw data-pictures/Fig 3G/psm3-2A/DIC.tif]

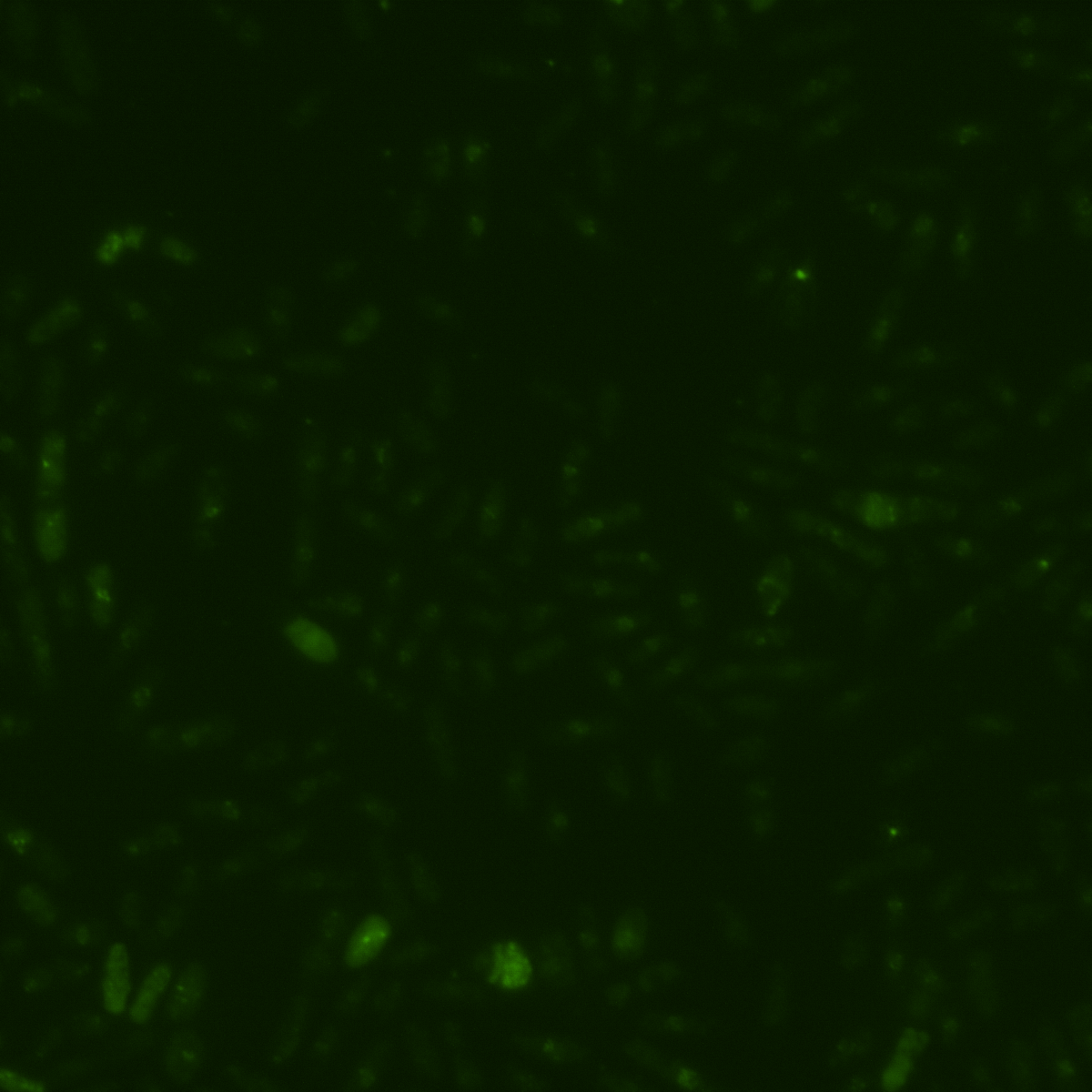

Supplement: Supplementary file 2 [file LSA-2023-02556_SdataF1.2_F2.2_F3.2_F4.2.zip › Raw data-pictures/Fig 3G/psm3-2A/Rad21-GFP.tif]

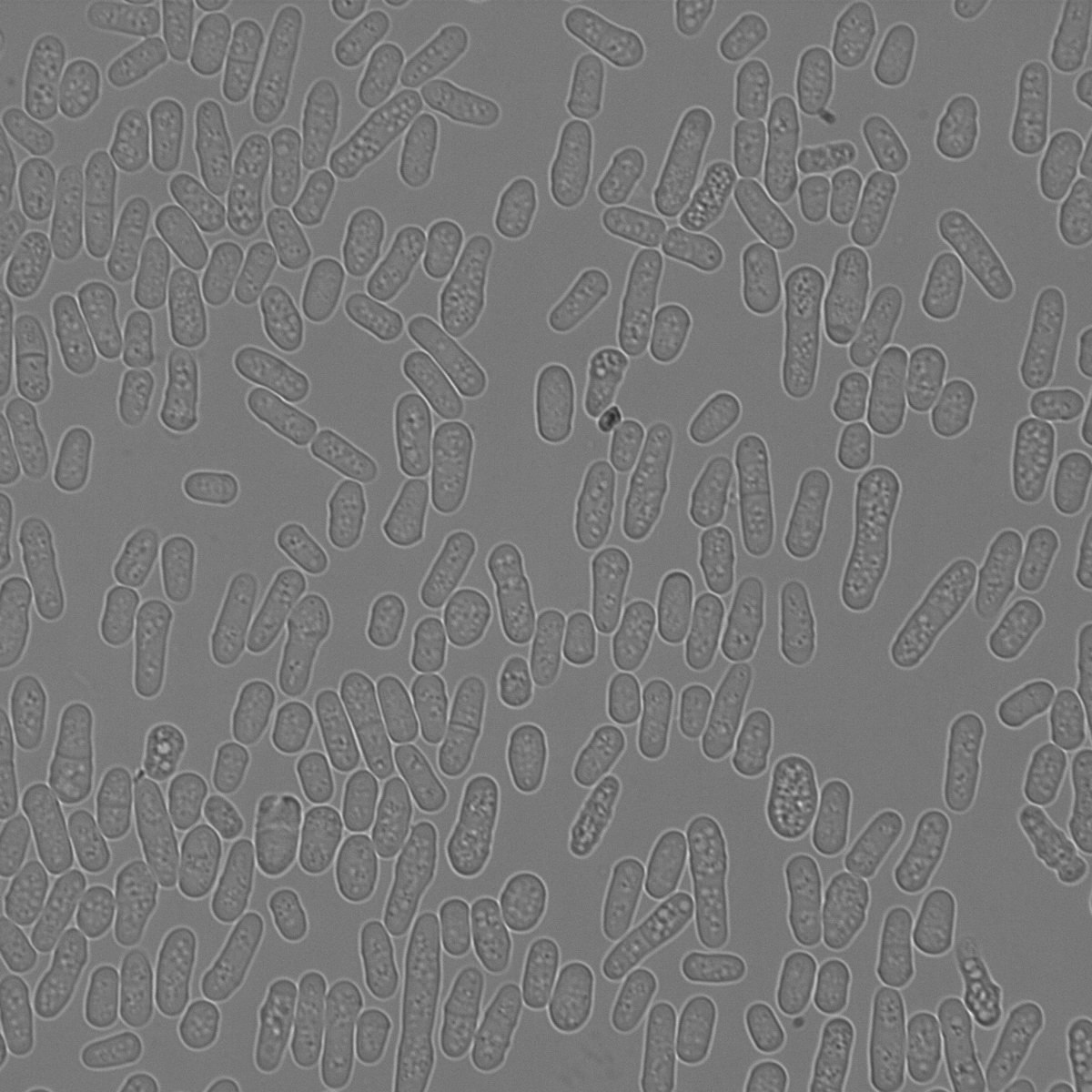

Supplement: Supplementary file 2 [file LSA-2023-02556_SdataF1.2_F2.2_F3.2_F4.2.zip › Raw data-pictures/Fig 3G/psm3-ED/DIC.tif]

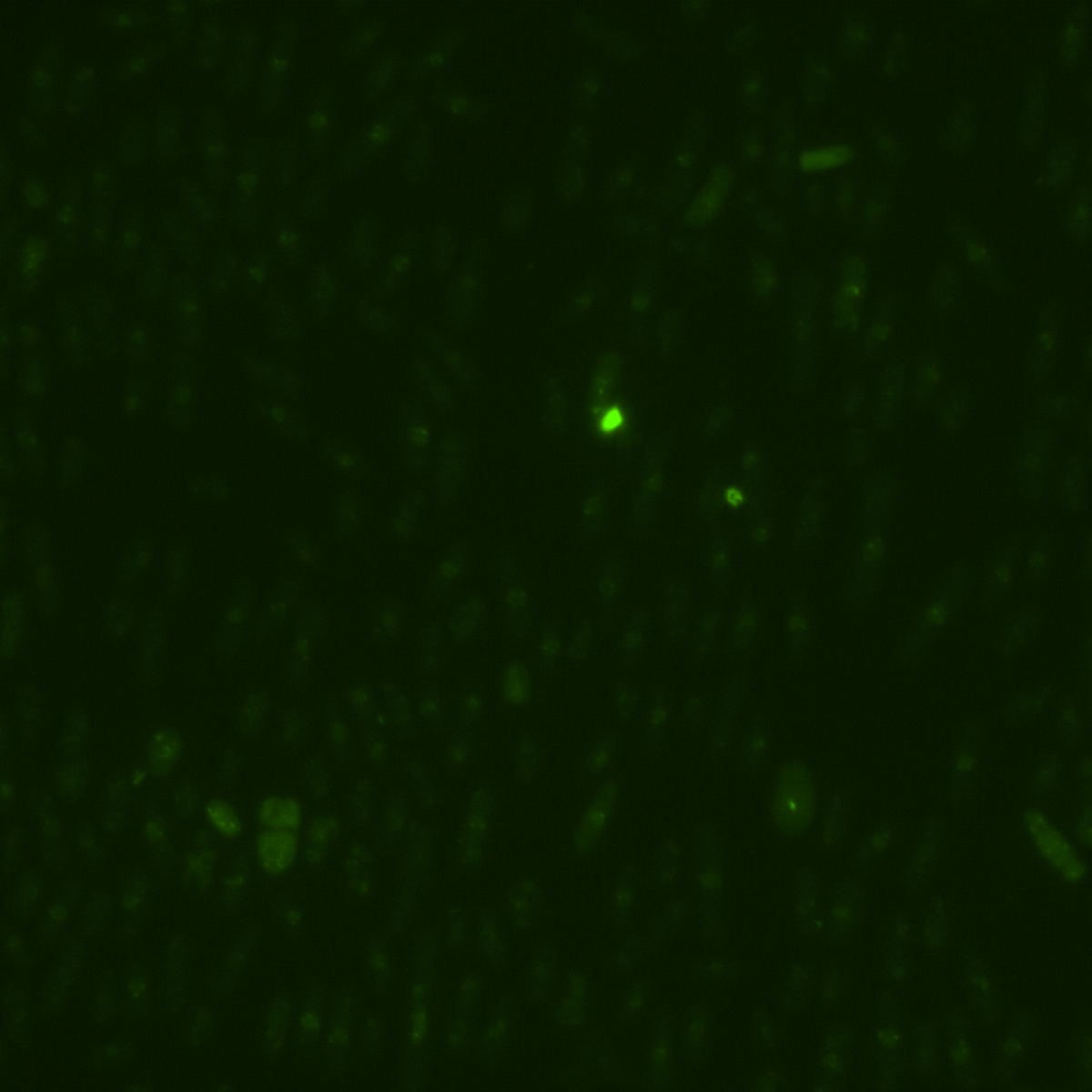

Supplement: Supplementary file 2 [file LSA-2023-02556_SdataF1.2_F2.2_F3.2_F4.2.zip › Raw data-pictures/Fig 3G/psm3-ED/Rad21-GFP.tif]

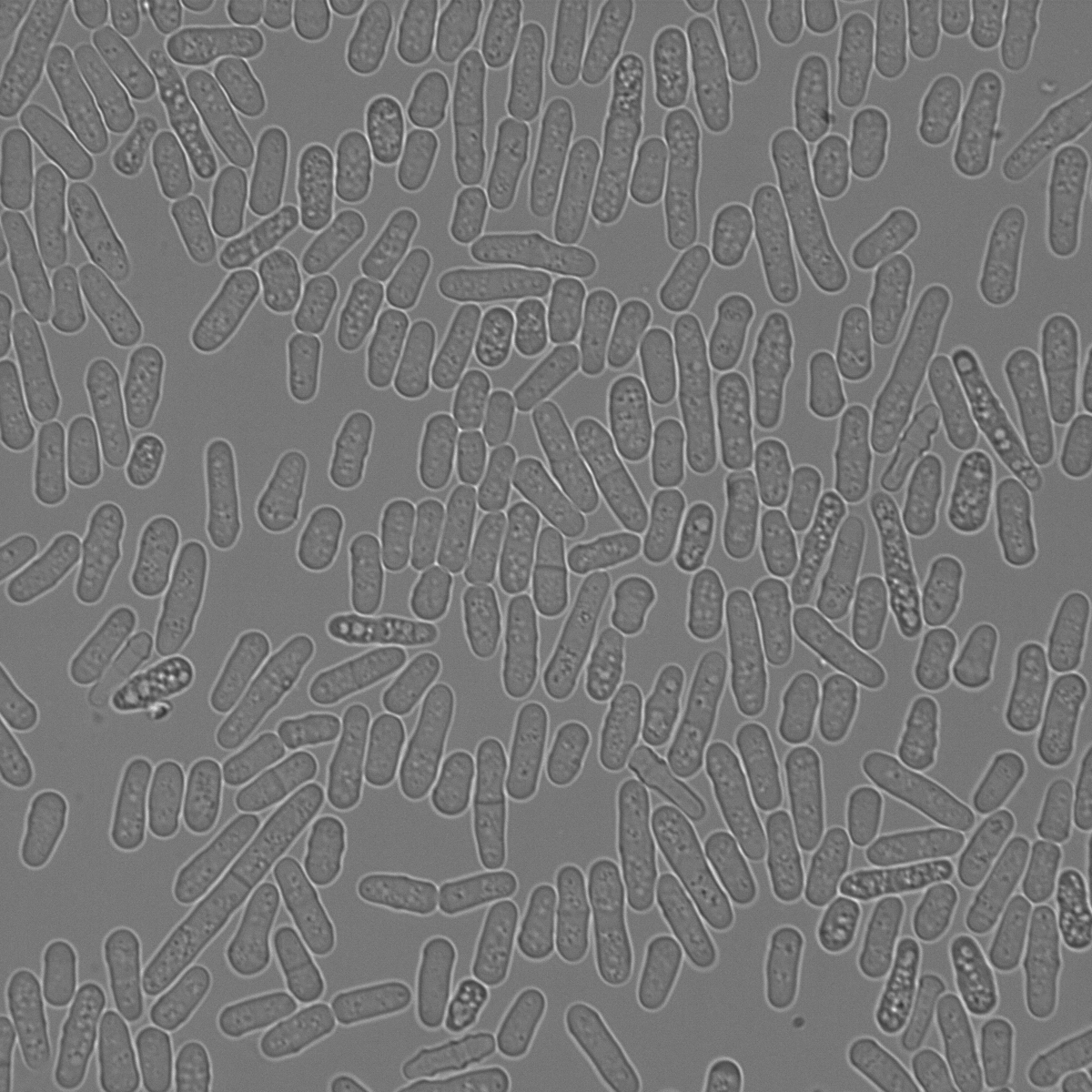

Supplement: Supplementary file 2 [file LSA-2023-02556_SdataF1.2_F2.2_F3.2_F4.2.zip › Raw data-pictures/Fig 3G/wt/DIC.tif]

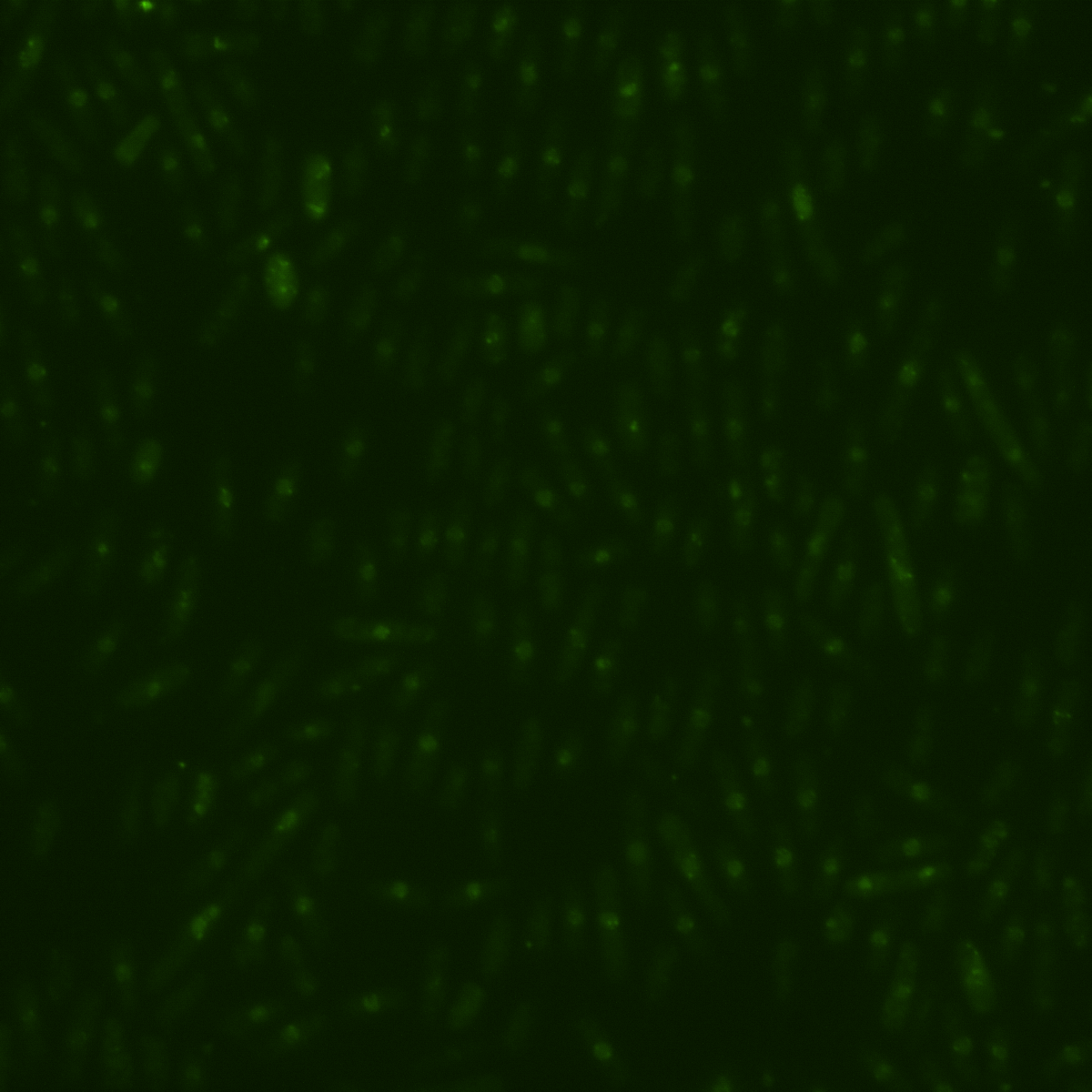

Supplement: Supplementary file 2 [file LSA-2023-02556_SdataF1.2_F2.2_F3.2_F4.2.zip › Raw data-pictures/Fig 3G/wt/Rad21-GFP.tif]

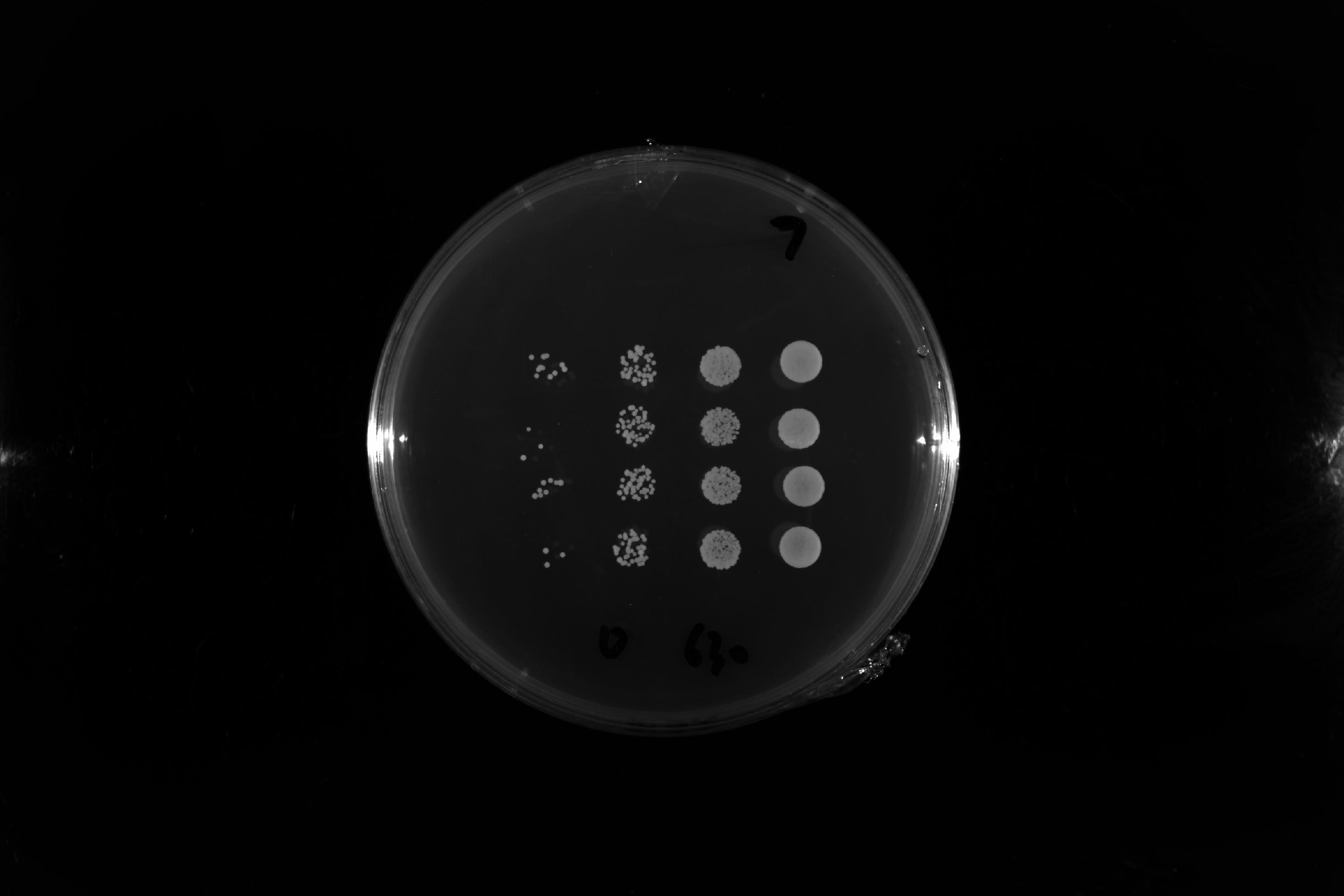

Supplement: Supplementary file 2 [file LSA-2023-02556_SdataF1.2_F2.2_F3.2_F4.2.zip › Raw data-pictures/Fig 3H/0 TBZ.tif]

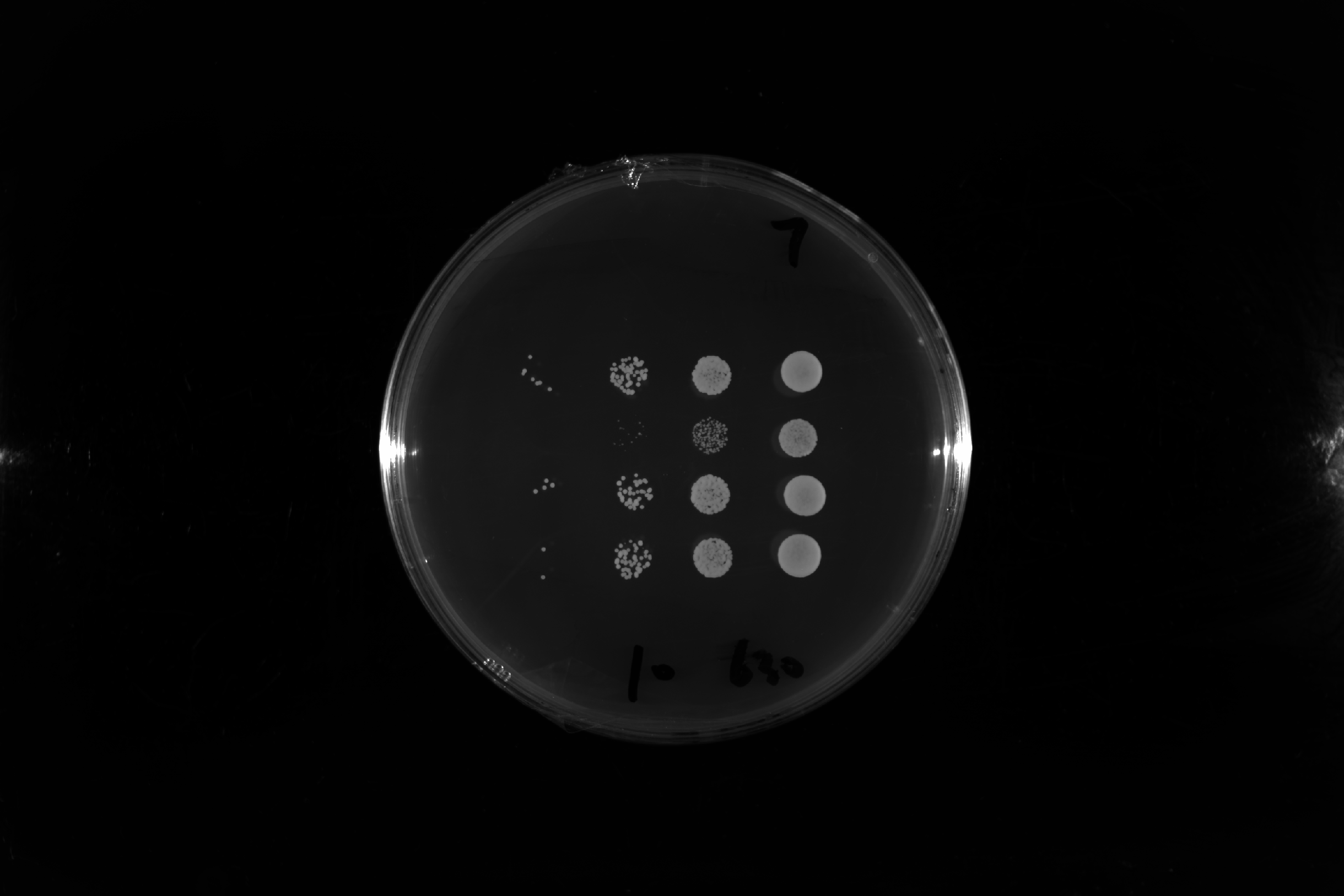

Supplement: Supplementary file 2 [file LSA-2023-02556_SdataF1.2_F2.2_F3.2_F4.2.zip › Raw data-pictures/Fig 3H/10 TBZ.tif]

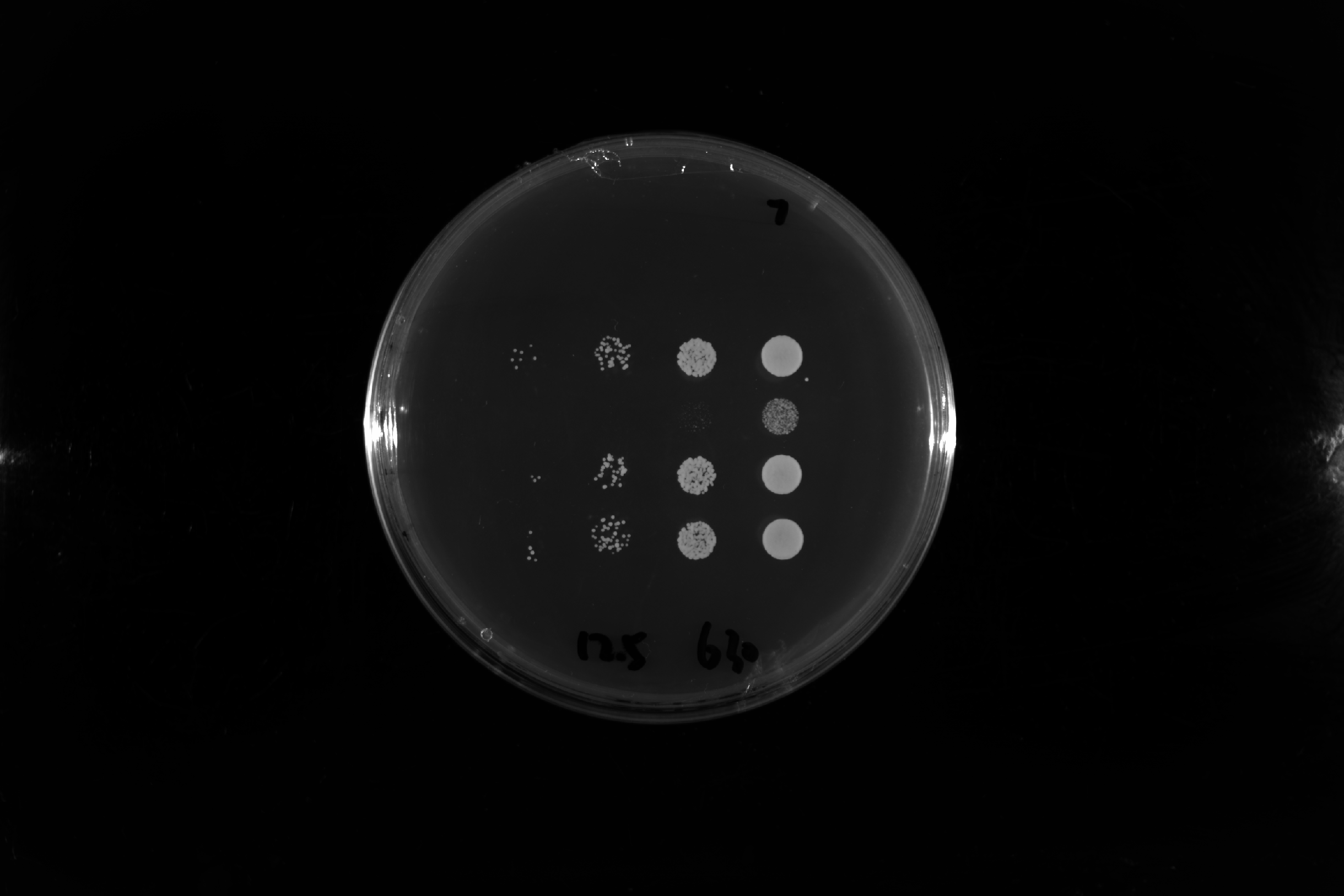

Supplement: Supplementary file 2 [file LSA-2023-02556_SdataF1.2_F2.2_F3.2_F4.2.zip › Raw data-pictures/Fig 3H/12.5 TBZ.tif]

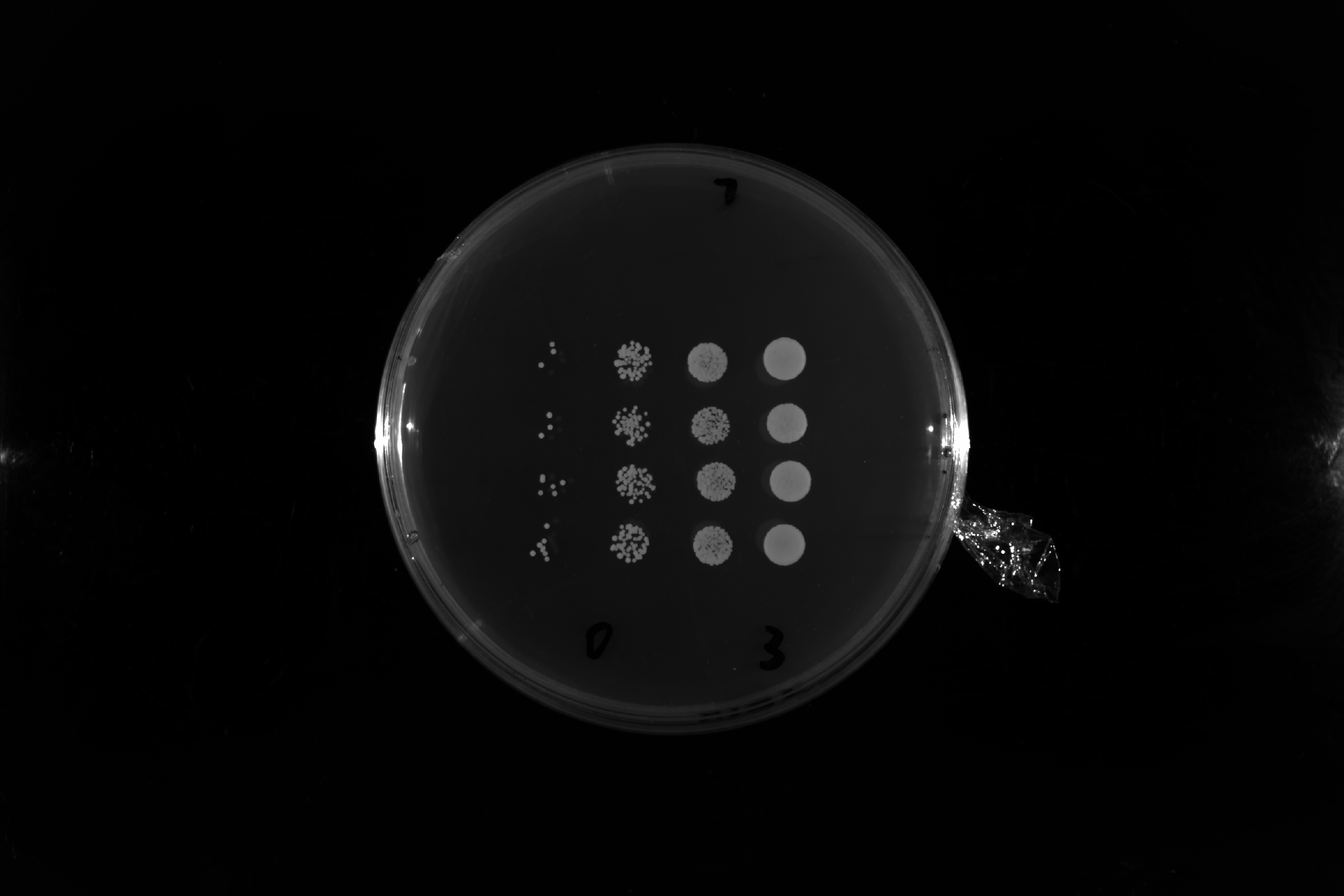

Supplement: Supplementary file 2 [file LSA-2023-02556_SdataF1.2_F2.2_F3.2_F4.2.zip › Raw data-pictures/Fig 4C/0 TBZ.tif]

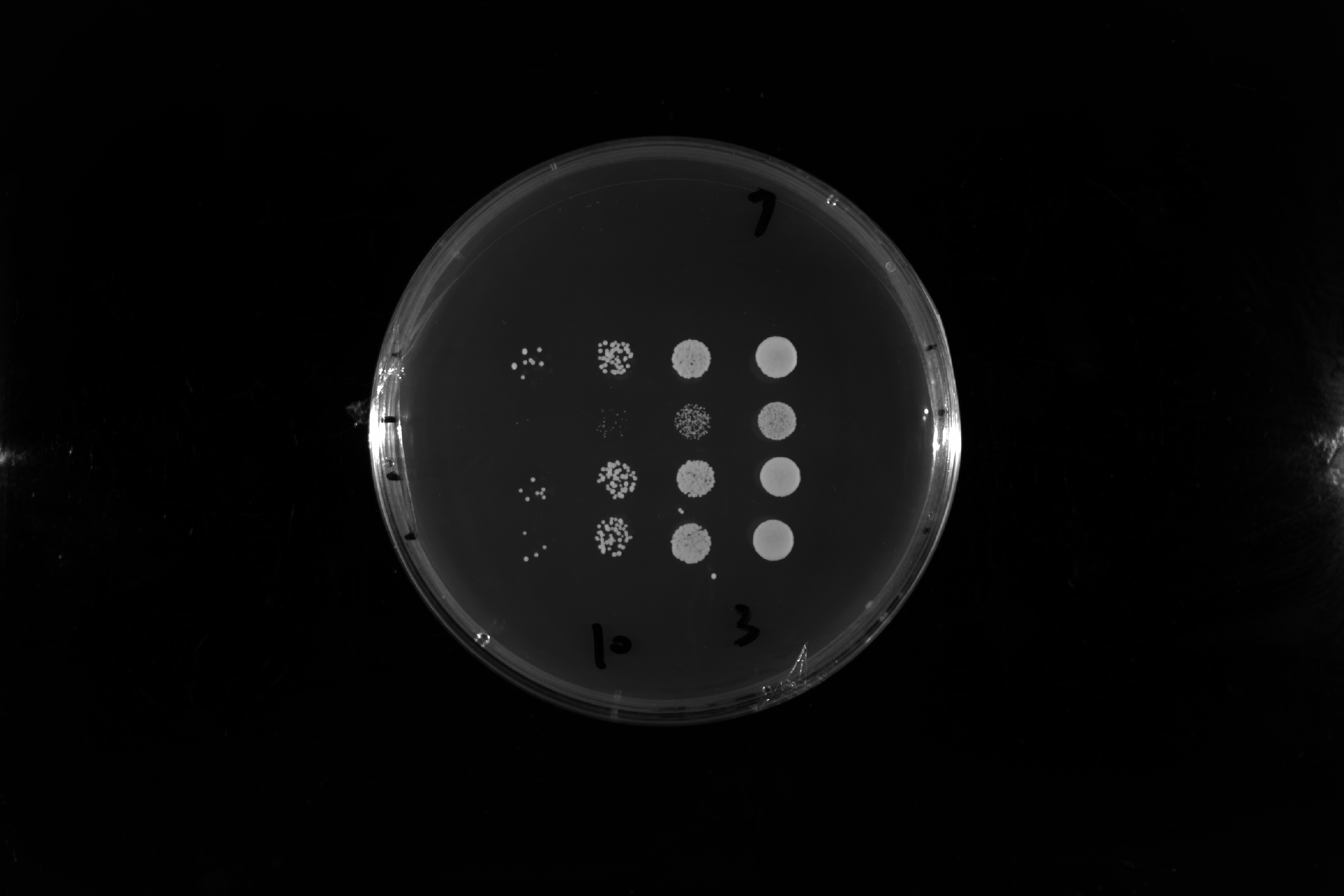

Supplement: Supplementary file 2 [file LSA-2023-02556_SdataF1.2_F2.2_F3.2_F4.2.zip › Raw data-pictures/Fig 4C/10 TBZ.tif]

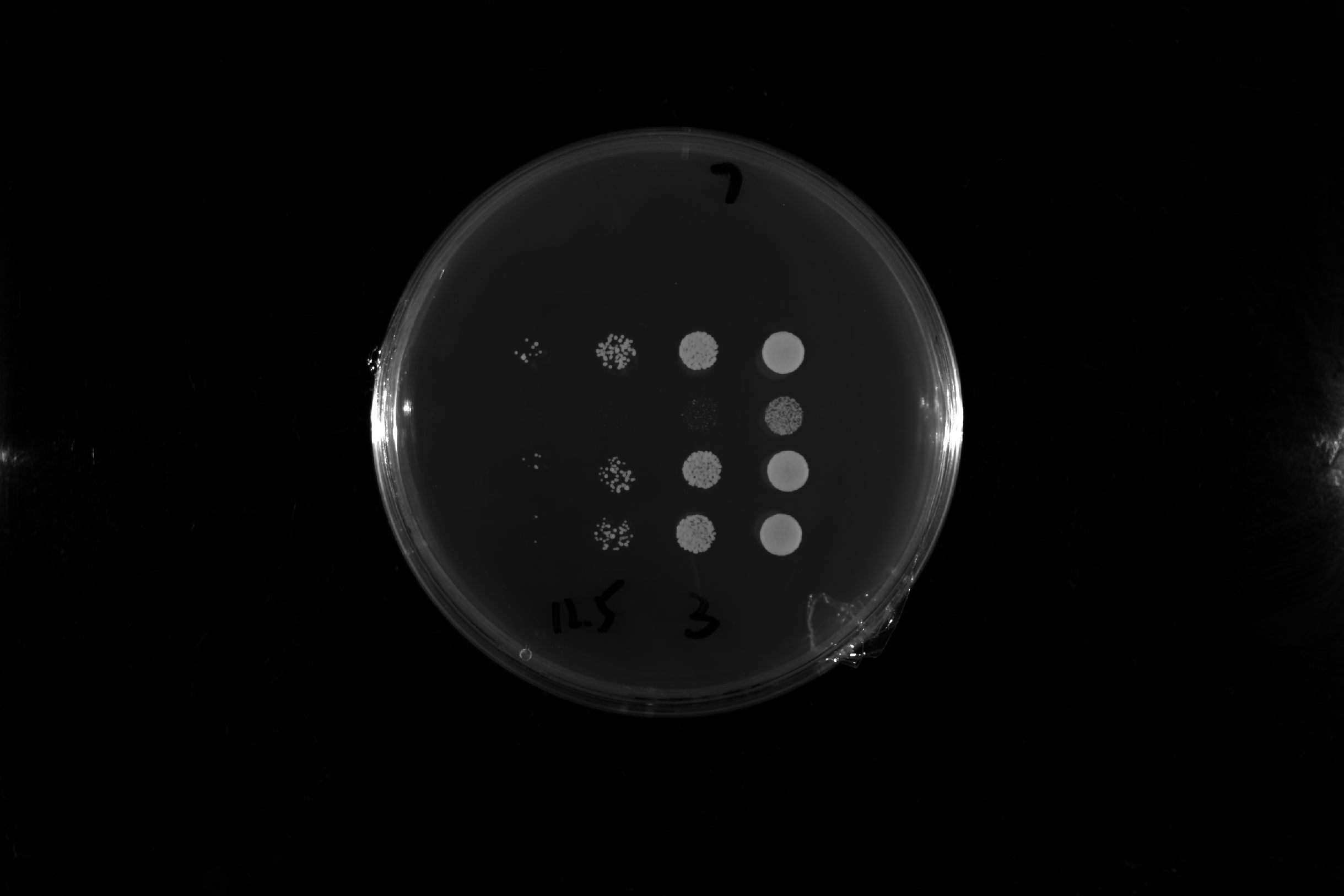

Supplement: Supplementary file 2 [file LSA-2023-02556_SdataF1.2_F2.2_F3.2_F4.2.zip › Raw data-pictures/Fig 4C/12.5 TBZ.tif]

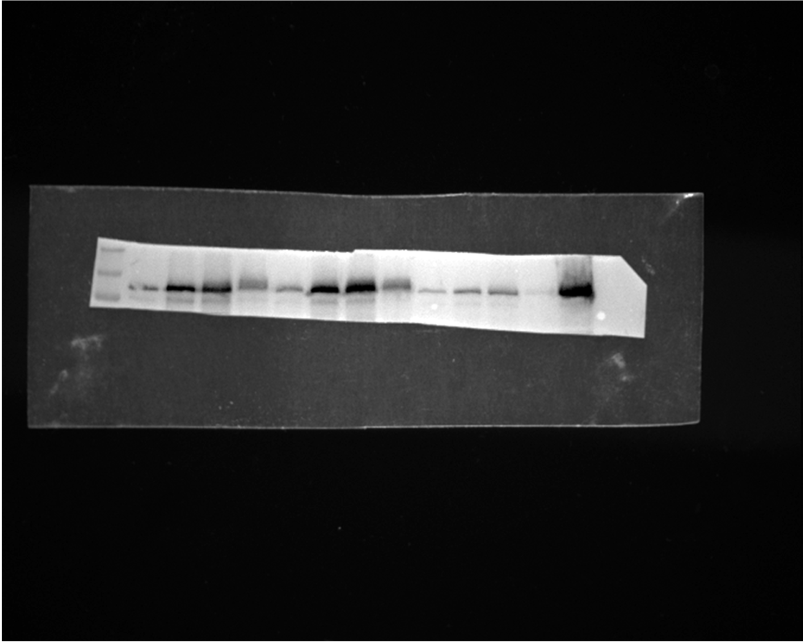

Supplement: Supplementary file 2 [file LSA-2023-02556_SdataF1.2_F2.2_F3.2_F4.2.zip › Raw data-pictures/Fig S4/Rec8-Myc.tif]

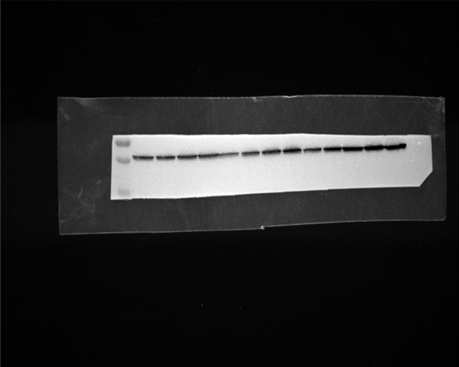

Supplement: Supplementary file 2 [file LSA-2023-02556_SdataF1.2_F2.2_F3.2_F4.2.zip › Raw data-pictures/Fig S4/Tublin.tif]
