# Supplementary material for: Phosphorylation of Rec8 cohesin complexes regulates mono-orientation of kinetochores in meiosis I
Source: Life Sci Alliance. 2024 Mar 6;7(5):e202302556. doi: 10.26508/lsa.202302556 (PMC10917647; doi:10.26508/lsa.202302556)
Supplement: Supplementary file 3 [file LSA-2023-02556_SdataF1.3.pptx]

## Slide 1
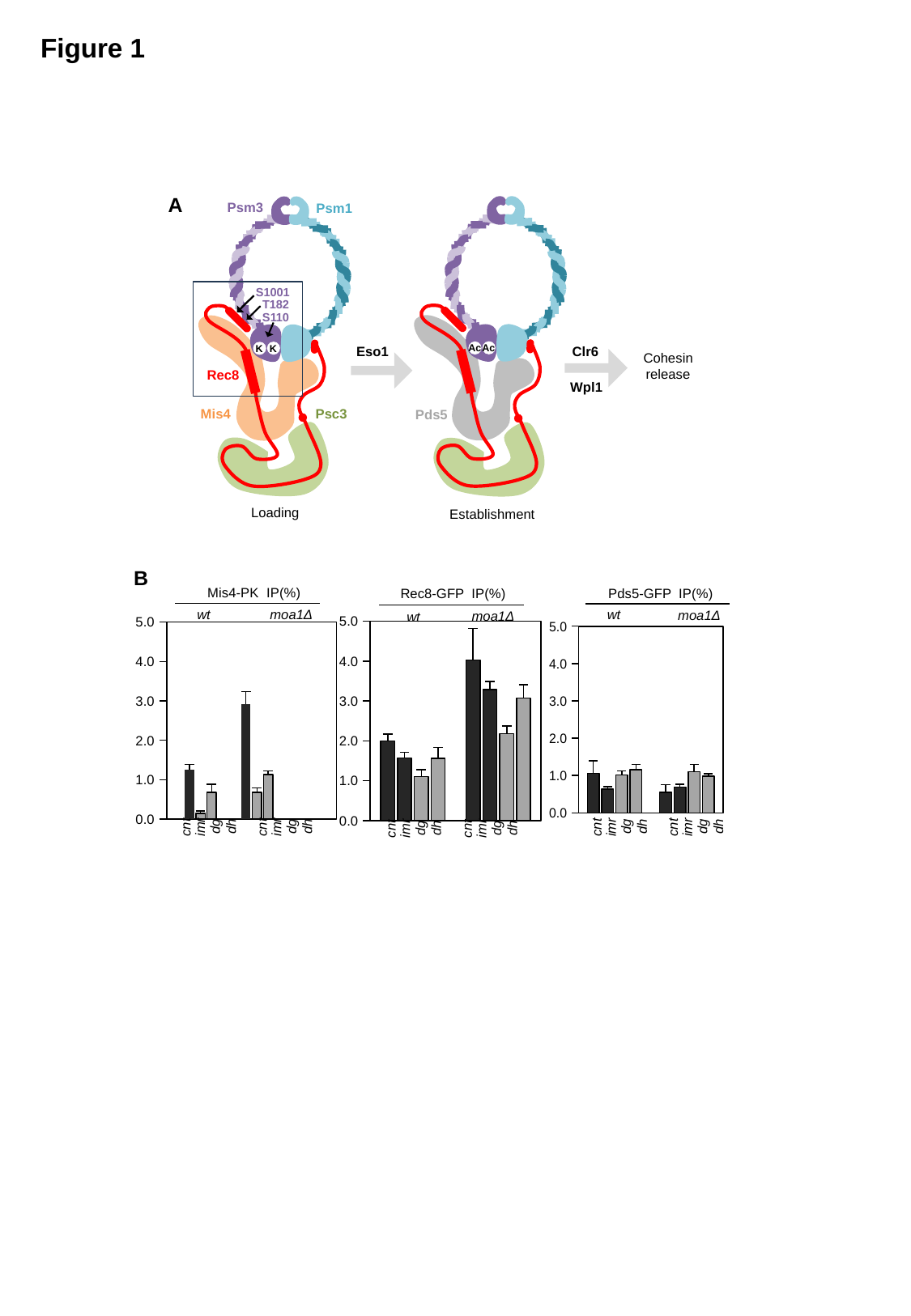

Figure 1
A
Psm3
Psm1
S1001
T182
S110
K
K
Eso1
Rec8
Psc3
Mis4
Loading
Ac
Ac
Pds5
Clr6
Cohesin release
Wpl1
Establishment
B
Mis4-PK IP(%)
wt
moa1Δ
### Chart
| Category | cnt | imr | dg | dh |
|---|---|---|---|---|
| D | 1.6220483745182435 | 1.2511617335282128 | 0.15020447911270032 | 0.6758910300663287 |
| C | 4.054001865162369 | 2.920835476957772 | 0.6812990043179316 | 1.130838192368071 |dh
dg
imr
cnt
dh
dg
imr
cnt
Pds5-GFP IP(%)
wt
moa1Δ
### Chart
| Category | cnt | imr | dg | dh |
|---|---|---|---|---|dh
dg
imr
cnt
dh
dg
imr
cnt
Rec8-GFP IP(%)
moa1Δ
wt
### Chart
| Category | cnt | imr | dg | dh |
|---|---|---|---|---|dh
dg
imr
cnt
dh
dg
imr
cnt
